# Pharmacodynamic interactions among meropenem ciprofloxacin and gentamicin in an in-vitro model

**DOI:** 10.1038/s41598-025-29354-y

**Published:** 2025-11-24

**Authors:** Zahra Sadouki, Emmanuel Q. Wey, Liam Read, Mark Bayliss, Alan Noel, Indran Balakrishnan, Timothy D. McHugh, Frank Kloprogge

**Affiliations:** 1 Institute for Global Health, University College London, London, UK; 2 Centre of Clinical Microbiology, University College London, Royal Free Campus, Rowland Hill Street, London, NW3 2PF UK; 3 Department of Infection, Royal Free London NHS Trust, London, UK; 4 North Bristol NHS Trust, Bristol, UK

**Keywords:** Meropenem, Gentamicin, Ciprofloxacin, Pharmacodynamics, *Escherichia coli*, Antibiotics, Pharmacodynamics

## Abstract

Meropenem, gentamicin and ciprofloxacin are often used together as empiric broad-spectrum therapy in different combinations. Recent regulations regarding the use of quinolones mean that greater justification is required when using this component of the combination. An understanding of the pharmacodynamic interactions of the different components of this combination is currently lacking—this could provide insight into the necessity of using the different agents. We aimed to study two- and three-way pharmacodynamic drug-drug interaction between meropenem, gentamicin and ciprofloxacin against *Escherichia coli*. Static time kill curve experiments were conducted with *Escherichia coli* (NCTC® 12,241) at 0.25–16 × MIC for a duration of 24 h with samples being collected at 0, 2, 4, 6, 8, and 24 h. Meropenem, gentamicin and ciprofloxacin were tested alone and in two- and three-way combinations. Bacterial load time series data were enumerated on Mueller Hinton agar and colony forming unit data were modelled using nonlinear mixed-effects models in the nlmixr package. Meropenem, gentamicin and ciprofloxacin two- and three-way combinations prevented regrowth, but regrowth was seen when these agents were studied alone. Gentamicin and meropenem were synergistic in decreasing ciprofloxacin IC_50_. The combination effects of meropenem and gentamicin and the addition of meropenem to a gentamicin/ciprofloxacin combination were indifferent. Our findings demonstrate no additional pharmacodynamic benefit of the meropenem/gentamicin/ciprofloxacin combination over gentamicin/ciprofloxacin. A quinolone free combination of meropenem/gentamicin prevented regrowth, it did not display further synergy on IC_50_ and was indifferent in initial killing rate.

**Supplementary Information:**

The online version contains supplementary material available at 10.1038/s41598-025-29354-y.

## Introduction

The combination of an aminoglycoside, beta-lactam and quinolone is not infrequently used to manage difficult infections, particularly in severe sepsis and is cited as one of the new recommendations with respect to MDR gram negative pathogens in the most recent surviving sepsis IDSA guidelines review^1^. Especially in complex infections where compartment pharmacokinetic/pharmacodynamic considerations apply, dual therapy may be required and confer a pharmacokinetic/pharmacodynamic benefit in the context of proven MDR gram negative infections in combination with definitive source control. Examples include a redox potential in abscesses (aminoglycosides), tissue penetration (bone and joint), devascularised tissues (Fournier’s gangrene and Necrotising fasciitis) and reduced anatomical compartment penetrations (CNS).

The pharmacological rationale for administering an antimicrobial drug combination is often based on increasing the spectrum of coverage. However, there are often pharmacodynamic interactions between the components that may improve the efficacy of the regimen.

In the three-way combination comprising meropenem, gentamicin and ciprofloxacin, meropenem lyses the bacterial cells by interfering with synthesis of peptidoglycan. This facilitates increased uptake of ciprofloxacin and gentamicin, thereby increasing bactericidal activity through prevention of DNA supercoiling and disruption of ribosomal protein synthesis respectively. The use of quinolones in this setting needs careful deliberation given recent MHRA regulations in response to safety concerns, as well as antibiotic stewardship considerations^2^.

The aim of this study was to describe pharmacodynamic drug-drug interactions for two- and three-way combinations of meropenem, gentamicin and ciprofloxacin against *Escherichia coli* (*E. coli*) through quantitative characterization using a factorial static time-kill approach and nonlinear mixed-effects modelling. The findings enhance our understanding of the mechanism of action of antimicrobial combination therapy and help inform rationalised use of such therapy. This will inform future research into antimicrobial combination therapy for Multi Drug Resistant (MDR) gram negative bacterial infections.

## Methods

### Static time-kill experiments

*Escherichia coli* (NCTC® 12,241) minimum inhibitory concentrations (MICs) were determined according to CLSI M100-ED32:2022 and were found to be in line with the published CLSI MIC QC ranges for non-fastidious organisms at 0.015 mg/L for ciprofloxacin, 1 mg/L for gentamicin and 0.03 mg/L for Meropenem.

Time-kill assays were conducted following CLSI M26-A guidelines using 96-well plates with a total volume of 200 μL. Serial dilutions in CAMHB were prepared for meropenem (Sigma, UK), ciprofloxacin (TOKU-E, US), and gentamicin (Sigma, UK) monotherapies and combination therapies. Concentrations ranged from 0.25 to 16 times the MIC, with two-way and three-way combinations adjusted proportionally. Plates were inoculated with 10^5^ CFU/mL *E. coli* and were incubated at 37.5 °C for 24 h. Bacterial counts (colony forming units, CFU) were performed hourly for the first 8 h, then at 24 h on Mueller–Hinton agar, with resistant subpopulations selected on agar supplemented with 2X and 8X MICs of each antibiotic. Data points represent the mean of biological duplicates and technical triplicates.

### Meropenem chemical degradation

Data from meropenem concentrations measured over a 24 h period was used to build a model that estimated the chemical degradation function of meropenem in Mueller Hinton broth at 37°C. Samples taken were stored at − 80°C and sent by courier on dry ice to the Bristol Antimicrobial Reference Laboratory (ARL).

Measurements of meropenem concentrations were conducted using a Shimadzu Prominence® (Kyoto, Japan) LC system coupled to a Sciex triple quadrupole 4000 QTRAP mass spectrometer (AB Sciex, Warrington, UK) operating in positive ion mode. Sciex Analyst v.1.5.1 software was used to control the LC–MS/MS system and collect the MS data.

LC/MS-grade formic acid, acetonitrile, methanol, and water were purchased from Fisher Scientific (Loughborough, UK). Meropenem Trihydrate was purchased from Sigma-Aldrich. Ertapenem (used as an internal standard) was purchased from Carbosynth (Berkshire, UK).

The samples stored at − 80°C were thawed and vortexed shortly before analysis. 20μL of each sample was dispensed into a 0.5 mL polypropylene Eppendorf® tube. The samples were then mixed thoroughly with 20μL of water and 40μL of 5 mg/L Ertapenem spiked in acetonitrile. After being left to stand at 4 °C for 10 min, the sample was centrifuged for 15 min at 17,000 g. 50μL of supernatant was then transferred to a glass HPLC vial containing 450μL of water. The final extract was thoroughly mixed prior to analysis. Then, the 2µL of the sample was injected and quantified using ertapenem as internal standard. The upper quantification limit was 1000 µg/mL and the lower quantification limit was 1 µg/mL.

Chromatographic separation was achieved using a Kinetex XB-C18 (Phenomenex ®) column (2.6µM 50X2.1mm ID, part no. 00B-4496-AN). The mobile phase consisted of 0.1% formic acid in water (eluent A) and acetonitrile (eluent B) operating in a binary step-wise gradient as follows: 2% eluent B—> 40% between 0.0 and 0.5 min, 40% B—> 95% between 0.5 and 1.0 min, 95% B between 1.0 and 1.5 min, dropping to 2% B at 1.6 min and re-equilibrating at 2% for 2.5 min. The MRM transitions for Meropenem and Ertapenem were set to m/z 384.2 → 254.0 and 476.1 → 432.1 respectively, with a dwell time of 20ms.

### ***Pharmacodynamic modelling***:

A nonlinear mixed effects model, built in nlmixr 2.0.7 using R 4.1.3, was used to describe the bacterial CFU/mL growth and killing dynamics time series data. Random effects (η) were distinguishing variability between experiments, departing from the population mean estimate (θ), rendering individual level parameter estimates (${P}_{i}$). Parameters with a biological lower limit of quantification at 0 were log-normally parameterised:

$${P}_{i}={e}^{\theta +\eta }$$

Residual variability (ε) within experimental time series data was additive to individual model predictions (IPRED) for the *i*^*th*^ experiments and *j*^*th*^ observation, after both-sided log-transformation:

$${Y}_{i,j}={IPRED}_{i,j}+\varepsilon$$

A linear meropenem degradation function was included for all meropenem containing regimens to characterise and embed the chemical degradation (${k}_{degradation}$) occurring at experimental conditions for meropenem concentrations (${C}_{MER}$).

$$\frac{d\left({C}_{MER}\right)}{dt}={k}_{degradation} \times {C}_{MER}$$

The structural PD model comprised a logistic growth model to embed biological plausibility.

$$\frac{d(B)}{dt}=\left({k}_{net}\times \left(1-\frac{B}{{10}^{{B}_{MAX}}}\right)\times B\right)$$

The parameters k_net_, B and B_max_ represented net growth rate, bacterial load and maximum carrying capacity. Low inoculum size, i.e. 10^3^ vs. 10^5^ cfu/ml, was tested as proportional covariate on k_net_ and B_max_. Drug parameters included maximum antibiotic effect (E_max_), half-maximal antibiotic effect (IC_50_), and a shape factor (γ) in the form of a logistic E_max_ model to parameterise bacterial killing. Emergence of bacterial regrowth was parameterised using a time dependent drug effect size parameter (β), and the duration of the treatment effect (τ). To characterise drug concentration dependency of bacterial regrowth emergence, drug effect was parameterised using linear or Emax models on parameters β or τ. For meropenem experiments C_drug_ was dynamically model predicted over time whilst for gentamicin and ciprofloxacin C_drug_ was constant.

$${Effect}_{drug}= {E}_{MAX}\times \frac{{{C}_{drug}}^{\gamma }}{{{IC}_{50}}^{\gamma }+{{C}_{drug}}^{\gamma }}\times \left(1-\beta \times \left(1-{e}^{-t\times \tau }\right)\right)$$

Drug effect was implemented into the differential equation as follow:

$$\frac{d(B)}{dt}=\left(\left({k}_{net}\times \left(1-\frac{B}{{10}^{{B}_{MAX}}}\right)-{Effect}_{drug}\right)\times B\right)$$

Drug interaction was tested as additivity.

$$Additivity={Effect}_{{Drug}_{A}}+{Effect}_{{Drug}_{B}}+{Effect}_{{Drug}_{C}}$$

The model was developed sequentially, first fitting growth control data only, followed by monotherapy, two-way combinations and finally three-way combinations. Parameters were fixed to estimates obtained earlier in the model development sequence in case of parameter instability due to increasing model complexity in the later stages of model development. Further synergy was evaluated using a forward stepwise inclusion method using *p* < 0.05 (ΔOFV = -3.84) for the inclusion of a drug presence, as categorical or continuous effect, on IC_50_, E_MAX_, β and τ as additional degree of freedom in a nested model. The ability of models to fit the time series data was further evaluated using goodness of fit plots^3^.

## Results

### Static kill curve experiment results

**Fig. 1:**
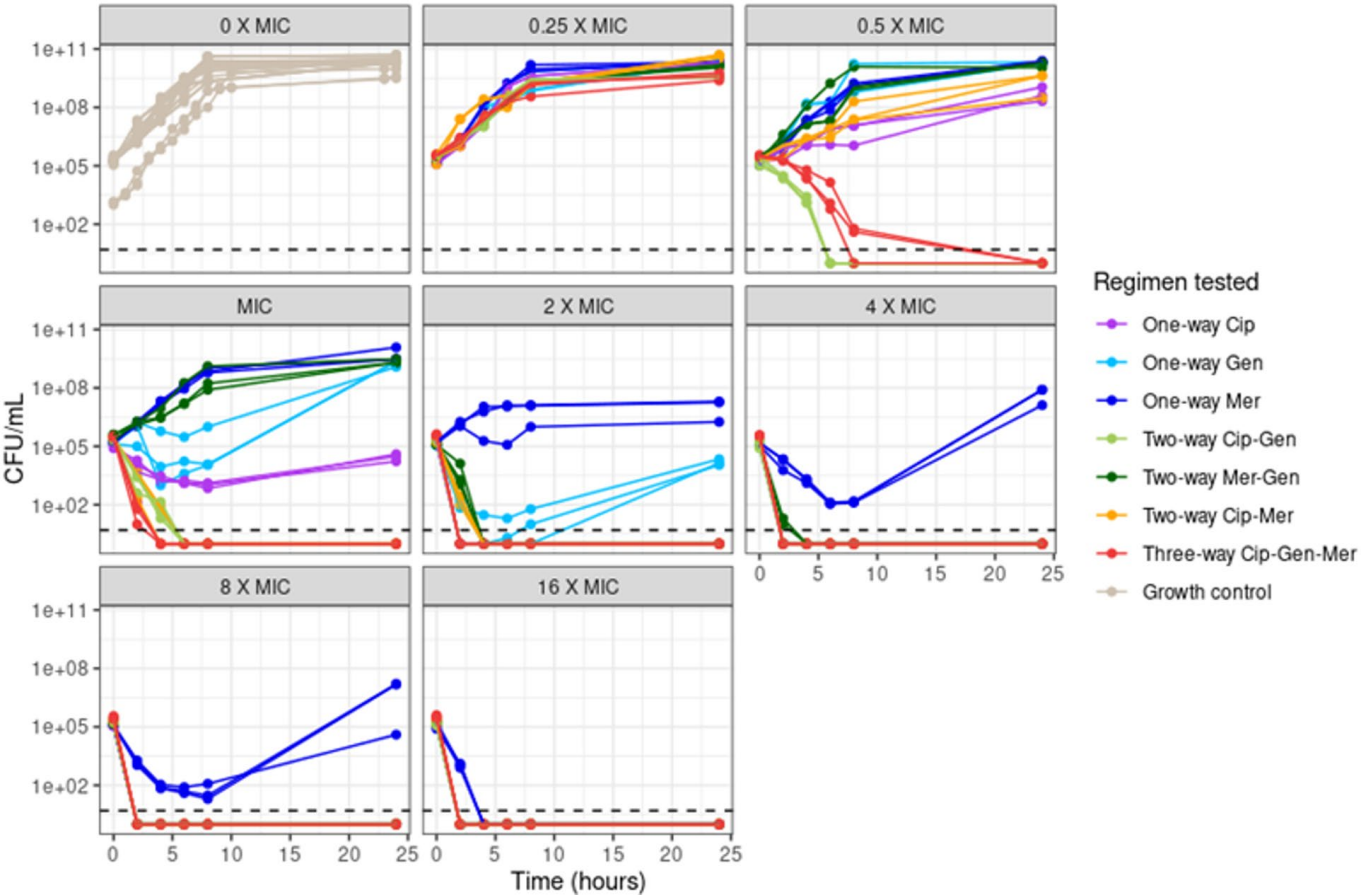
Static time kill curves of meropenem (Mer), ciprofloxacin (Cip) and gentamicin (Gen) antibiotic combinations. CFU/mL on the y-axis over 24 h on the x-axis for NCTC® 12,241 *E. coli* against concentration ranges from 0.25 times MIC to 16 times MIC. Data are stratified by multiplicities of MIC tested (panel) and by combination tested (colour). Biological repeats are plotted independently, and each data point represents the geometric mean of three technical repeats. Antibiotic free growth control represented as 0 X MIC panel (top left). Mer, cip and gen MIC’s were 0.03, 0.015, and 1 mg/l.

Figure 1 displays the *in-vitro* killing dynamics from time kill experiments for ciprofloxacin, gentamicin and meropenem as monotherapy, two-way and three-way combinations against *E. coli* (NCTC® 12,241). Data were stratified by MIC, i.e. 0.03, 0.015, and 1 mg/l for meropenem, ciprofloxacin and gentamicin, to dissect killing dynamics between the different regimens.

Different inoculum sizes were studied for the drug free experiments to provide a heterogenous range of growth data (Fig. 1). Maximum carrying capacity at 10^11^ CFU/mL was somewhat lower for lower inoculum size experiments, i.e. low and high inocula of 10^3^ and 10^5^ CFU/mL. No difference in killing dynamics was observed at the highest and lowest multiples of MIC, i.e. 16 × and 0.25 × MIC. Ciprofloxacin-gentamicin, as two-way combination, displayed the same antimicrobial effect compared to the meropenem-ciprofloxacin-gentamicin three-way combination, with all multiples, except 0.25 × MIC, producing killing below Limit Of Detection (LOD). The other two-way combinations, i.e. meropenem-gentamicin and ciprofloxacin-meropenem, required ≥ 2 × MIC to achieve comparable killing to ciprofloxacin-gentamicin (Fig. 1 and Suppl Fig. 1).

### Quantifying growth and killing

Differences in killing dynamics were investigated in detail by using nonlinear mixed-effects modelling to dissect and quantify pharmacodynamic drug-drug interactions and determine antagonistic and synergistic effects. The developed model was adequately describing the observed data (Suppl. Figure 2).

Growth control time series data from 24 experiments were fitted using a logistic mixed-effects model with random effects on variability between experiments and residual effect on variability within experiments (Table 1). The effect of inoculum size (i.e. 10^3^ or 10^5^ CFU/ml) on structural model parameter B (inoculum size) and B_MAX_ (maximum carrying capacity) significantly improved the model fit whilst the structural model parameter k_net_ (net bacterial growth) remained unaffected.

**Table 1:** Final parameter estimates of the pharmacodynamic model.

Model	Parameter	Model estimate (%RSE)	BSV*
Growth	*Growth model parameters*		
	k_net_	1.35 (7.91)	12.9
	B_0_ (CFU/ml)	5.29 (0.478)	
	B_MAX_ (CFU/mL)	10 (0.431)	
	*Innoculum effect on growth model parameters*		
	Categorical effect low inoculum—B_0_	− 0.326 (6.75)	
	Categorical effect low inoculum—B_MAX_	− 0.11 (14.7)	
Meropenem	Bacterial killing parameters		
	E_MAX_	4.18 (3.76)	5.43
	IC_50_ (mg/L)	0.0781 (9.5)	72.3
	hill	2.76 (31.2)	26.4
	*Emergence of bacterial regrowth parameters*		
	BETA	0.922 (28.7)	1.58
	TAU	0.570 (*fixed*)	196
	*Proportional effect MER concentration on bacterial regrowth parameter TAU*		
	Coefficient (change per mg/L MER)	0.00155 (39.9)	
Gentamicin	Bacterial killing parameters		
	E_MAX_	5.47 (1.32)	
	IC_50_ (mg/L)	1.12 (64.5)	25.5
	hill	3.63 (12.8)	12.8
	*Emergence of bacterial regrowth parameters*		
	BETA	0.829 (*fixed*)	0.475
	TAU	0.517 (*fixed*)	146
	*E*_*MAX*_* model for GEN concentration on bacterial regrowth parameter BETA*		
	IC_50_ (mg/L)	5.72 (*fixed*)	
	E_MAX_	− 2.97 (*fixed*)	
	hill	20 (*fixed*)	
Ciprofloxacin	*Bacterial killing parameters*		
	E_MAX_	4.55 (4.69)	37.6
	IC_50_ (mg/L)	0.0106 (1.79)	8.58
	hill	3.58 (12.4)	12.1
	*Emergence of bacterial regrowth parameters*		
	BETA	0.674 (*fixed*)	1.67
	TAU	0.359 (*fixed*)	192
	*E*_*MAX*_ *model for CIP concentration on bacterial regrowth parameter BETA*		
	IC_50_ (mg/L)	0.017 (*fixed*)	
	E_MAX_	− 4 (*fixed*)	
	hill	20 (*fixed*)	
Drug interactions	*Categorical effect for any 2- or 3- way combination on bacterial regrowth parameter BETA*		
	Coefficient	− 1 (*fixed*)	
	*Proportional effect for presence of MER or GEN on CIP bacterial killing potency parameter IC*_50_		
	Coefficient MER presence on IC_50_CIP_	− 0.353 (*fixed*)	
	Coefficient GEN presence on IC_50_CIP_	− 0.576 (*fixed*)	
Residual variability	Additive on logarithmic data	0.864	

*BSV = random variability between experiments, expressed as %CV apart for BETA which were expressed as additive eta estimates.

MER: meropenem, GEN: gentamicin, CIP: ciprofloxacin, knet: net growth, B_0_: inoculum size, B_MAX_: Maximum carrying capacity, E_MAX_: maximum effect, IC_50_: half-maximum effect, hill: shape factor, BETA: maximum loss of antimicrobial effect, TAU: shape factor loss of antimicrobial effect in time and cov: covariate effect relation between confounder and parameter.

**Fig. 2:**
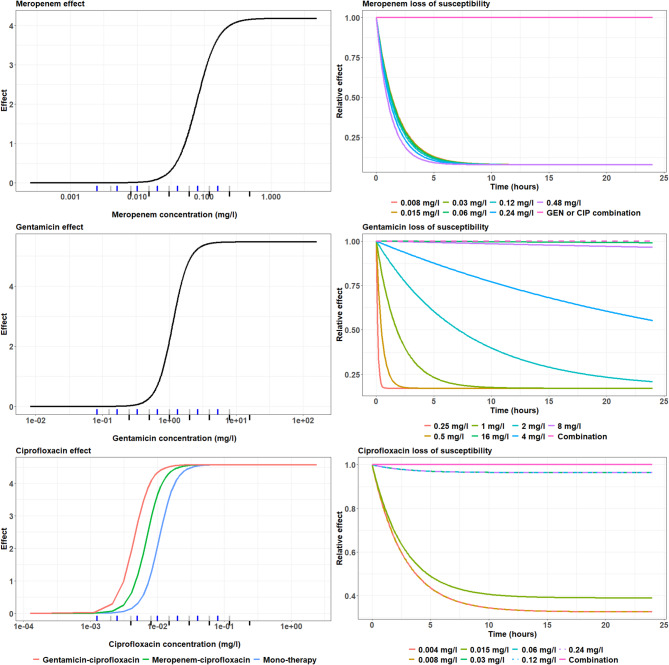
Bacterial killing effect and emergence of bacterial regrowth for meropenem, gentamicin and ciprofloxacin containing regimens. Figures on the left represent concentration-effect curves to show the killing effect observed (y-axis) against the scaled antibiotic concentration (x-axis). The markings on the x-axis represent the concentrations tested, i.e. one-way regimen in black, two-way regimen in grey, and three-way regimen in blue. Figures on the right represent emergence of bacterial regrowth through the decline of killing effect in time which was presented as relative effect (y-axis) over time (x-axis).

Chemical degradation was accounted for in meropenem concentration calculations as we found that meropenem concentrations at 37 °C dropped by 10% in 8 h; this degradation was extrapolated for the 24-h duration of our static kill curve experiment (Suppl. Figure 3). Meropenem E_MAX_ (Maximum effect) was estimated at 4.18 whilst IC_50_ (concentration causing half-maximum effect) was estimated at 0.0781 mg/L (Table 1) with tested concentrations covering the critical points in the concentration-effect curve (Fig. 2). Unlike for gentamicin and ciprofloxacin there was little spread in correlation between meropenem exposure and time dependent drug effect, which was captured using a proportional relationship between meropenem exposure and τ; consequently only 16 × MIC (0.48 mg/L) meropenem prevented regrowth (Figs. 1 and 2 and Table 1).

Gentamicin tested concentrations covered the critical points in the concentration-effect curve (Fig. 2) and E_MAX_ was estimated at 5.47 whilst IC_50_ was estimated at 1.12 mg/L (Table 1). Gentamicin killing could be sustained, without presence of waning, for the highest exposures (Fig. 1) whilst the equivalent of 8 and 16 × MIC produced a relative effect of 0.5 and near 1 at 24 h compared to baseline (Fig. 2). The gentamicin concentration dependent time effect was characterised using a logit type E_MAX_ relationship on β with further flexibility embedded for the lower gentamicin exposures using an inverse relationship on τ, i.e. $\tau \times \frac{1}{{C}_{MER}}$. (Table 1).

Amongst all three drugs in mono-therapy ciprofloxacin displayed most consistent killing with little presence of a waning effect over time (Fig. 1). E_MAX_ (Maximum effect) was estimated at 4.55 whilst IC_50_ (concentration causing half-maximum effect) was estimated at 0.0106 mg/L (Table 1); tested concentrations covered the critical points in the concentration-effect curve (Fig. 2). Cessation of ciprofloxacin drug effect did not occur at concentrations > 1 × MIC (Fig. 1) and the ciprofloxacin concentration dependent time effect was characterised using a logit type E_MAX_ relationship on β (Table 1).

### Quantifying pharmacodynamic drug-drug interactions

All tested drugs in combination, whether in two- or three-way, resulted in sustained killing during the 24-h experiment without evidence of regrowth, unlike the effect seen with mono-therapy at the investigated concentration range (Table 1 & Figs. 1 and 2). This was implanted as categorical effect on bacterial regrowth parameter BETA.

The combined gentamicin-ciprofloxacin effect was additive with a synergistic proportional effect of gentamicin presence on ciprofloxacin IC_50_, resulting in a 57.6% lower value (Table 1). This resulted in greater activity at 0.5 and 1 × MIC when gentamicin-ciprofloxacin was given in combination compared with either agent alone (Fig. 1). Addition of meropenem, i.e. the three-way combination of meropenem-gentamicin-ciprofloxacin, resulted in a similar killing trajectory compared to the two-way gentamicin-ciprofloxacin combination, with the exception of 0.5 and 1 × MIC (Fig. 1). Adding meropenem as additive drug effect on top of the gentamicin-ciprofloxacin two-way combination did not yield an improvement of the model fit, indicating the interaction was indifferent.

The combined meropenem-ciprofloxacin effect was additive with a synergistic effect of meropenem presence on ciprofloxacin IC_50_, resulting in a 35.3% lower value (Table 1). At 1 and 2 × MIC, this yielded antimicrobial effects of meropenem and ciprofloxacin that were greater in combination compared to when they were given alone (Fig. 1).

No additional antimicrobial effects were observed when meropenem and gentamicin were given in combination, compared to when gentamicin was given alone. Apart from the 1 × MIC concentration, the meropenem-gentamicin combination experiment followed the same killing trajectory up to the point of regrowth for monotherapy.

## Discussion

Here we dissected the role of each antibiotic in the meropenem-gentamicin-ciprofloxacin combination regimen.

Gentamicin has been reported to exhibit synergistic activity with cell-wall acting antibiotics^4–7^, but we only found that the meropenem-gentamicin two-way combination reversed waning of the antimicrobial effect over time while no synergy was observed in the cidal effects (Fig. 2). However, others have reported up to fivefold reductions in MIC (from 64 to 2 mg/L) for carbapenem and gentamicin combinations^4^. Amikacin and meropenem in combination have also been reported synergistic, through a reduced MIC and increased capacity to reduce biofilm formation^8,9^.

The time-kill experiments presented here have enabled us to disentangle early cidal effects from regrowth; this cannot be done by MIC experiments only. Given that our results show no regrowth in the presence of the meropenem-gentamicin two-way combination at the tested concentration range our findings are in line with previous reports (Fig. 2). Moreover, colleagues often reported synergy in MDR bacteria, with mono-resistance to the drugs investigated in combinations, whereas this investigation was conducted using a sensitive laboratory strain^6,9–12^.

Incorporation of ciprofloxacin in the regimen results in increased pharmacodynamic efficacy as combination with either meropenem or gentamicin reduces the ciprofloxacin’s IC_50_ (Fig. 2). This is consistent with literature in which meropenem and ciprofloxacin showed synergy against 18/52 Acinetobacter baumannii strains^12^. The three-way drug combinations did not display greater antimicrobial effects compared to any of the two-way combinations when challenging the susceptible laboratory stain *E. coli* NCTC 12,241.

The inoculum effect we found is in line with other reports (Fig. 2). Brook et al. reported in 1989 that bacterial susceptibility to a range of antibiotics is reduced when the inoculum is increased^13^. Likewise, meropenem chemical degradation was in line with reports of meropenem being unstable in solution due to degradation of the β-lactam ring (Suppl. Figure 3). Others reported varying rates of degradation ranging from 90% in 5.7 h to 20% after 24 h^14–16^.

The limitations of this study are; experiments were conducted using NCTC® 12,241 *E. coli*, and pharmacodynamic drug-drug interactions were characterised based on static time-kill experiments that followed an experimental setup based on the DiaMOND assay^17^. The choice for a susceptible laboratory strain limits the generalisability of the results to commonly encountered clinical isolates including quinolone-resistant and ESBL-producing *E. coli*. Nevertheless, the study enabled obtaining an understanding on the pharmacodynamic drug-drug interactions which could inform future research into antimicrobial combination therapy for Multi Drug Resistant (MDR) gram negative bacterial infections.

Furthermore, the experimental design only allowed evaluation of pharmacodynamic interactions at antimicrobial combination exposures at fixed 1:1:1 ratio. This may not necessarily reflect the *in-vivo* effects given the influence of the biological behaviour, whilst leaving out the immune system. Moreover, potential asymmetries in combinatorial effects may not be adequately captured. The approach has however been used for efficiently measuring high-order drug interactions with other bacteria^17^.

In conclusion, all two- and three-way combinations of meropenem, gentamicin and ciprofloxacin prevent regrowth as opposed to when these components were studied on their own. Ciprofloxacin antimicrobial effects improved in combination with either meropenem or gentamicin through reduced ciprofloxacin IC_50_. The combination effects of meropenem and gentamicin were indifferent as was the addition of meropenem to a gentamicin/ciprofloxacin combination.

In light of the recent move towards reduced use of quinolones^2^ our findings emphasize the added value of a quinolone in the drug combination. On the other hand, a quinolone free combination of meropenem and gentamicin did prevent regrowth, it just did not display further synergy on IC_50_ and was indifferent, in contrast to the reductions in ciprofloxacin IC_50_ seen when meropenem and gentamicin were added to ciprofloxacin containing combinations.

## Supplementary Information

Below is the link to the electronic supplementary material.

Supplementary Material 1

## Data Availability

All data is available upon reasonable request at f.kloprogge@ucl.ac.uk.
