# Supplementary material for: Pharmacodynamic interactions among meropenem ciprofloxacin and gentamicin in an in-vitro model
Source: Sci Rep. 2025 Nov 24;15:45244. doi: 10.1038/s41598-025-29354-y (PMC12749025; doi:10.1038/s41598-025-29354-y)
Supplement: Supplementary file 1 — Supplementary Material 1 [file 41598_2025_29354_MOESM1_ESM.docx]

**Suppl Fig 1: Static time kill curves of meropenem (Mer), ciprofloxacin (Cip) and gentamicin (Gen) antibiotic combinations.**


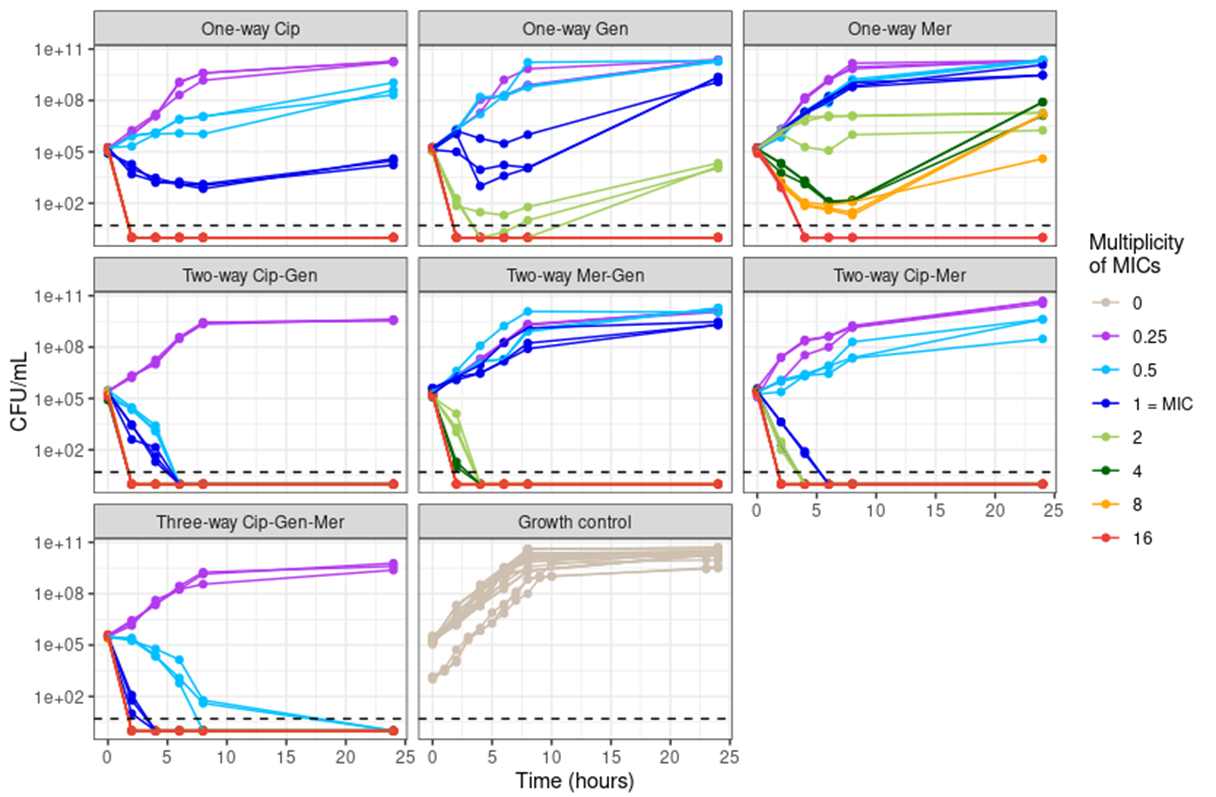


CFU/mL on the y-axis over 24 hours on the x-axis for NCTC® 12241 *E. coli* against concentration ranges from 0.25 times MIC to 16 times MIC. Data are stratified by antibiotic alone or in combination (panel) and by MIC multiplicity (colour). Biological repeats are plotted independently, and each data point represents the geometric mean of three technical repeats. Mer, cip and gen MIC’s were 0.03, 0.015, and 1 mg/l.

**Suppl Fig 2: Goodness of fit plots displaying observed bacillary load against population predicted (left) and individual predicted (right) bacillary load.**

The y-axis in both figure panels represent observed bacillary load measurements. The x-axis in the left-panel represents population predicted bacillary load measurements and in the right-panel individual predicted bacillary load measurements. The red solid line represents the unity line. Black and red dots represent observed and censored bacillary load measurements.

| Suppl Fig 3: Meropenem degradation over time (hours) for observations (DV) individual predictions (IPRED) and population predictions (PRED) |
| --- |
| **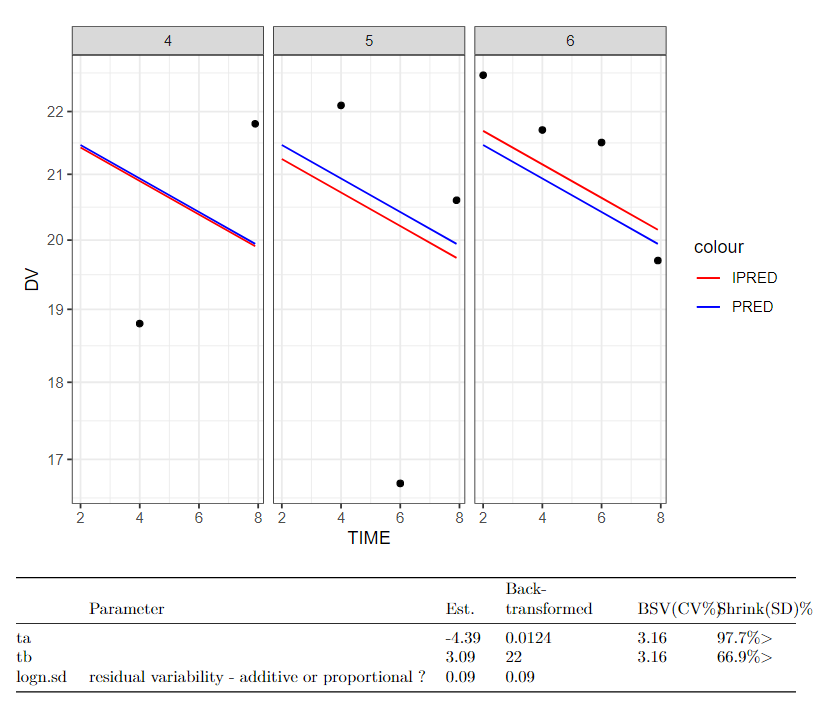** |

Figure panels on the top represent meropenem concentration (y-axis) over time (x-axis) profiles for three experimental replicates. Black dots represent observed concentrations whilst the red and blue lines represent individual and population model predicted meropenem concentrations. The table at the bottom is a summary of the linear mixed-effects model parameter estimates. The baseline estimate is represented by tb, with the slope being ta. Back-transformed estimated represent the mean estimate in the population with BSV representing log-normally distributed random variability between experiments departing from the population mean. Shrinkage on random variability is presented as %SD.
